# Calendar

**DOI:** 10.1155/S1463924685000475

**Published:** 1985


					Journal of Automatic Chemistry ofClinical Laboratory Automation, Vol. 7, No. 4 (October-December 1985), p. 208
Calendar
Further information from Ms Shirley
Schlessinger, Symposium Manager of
HPLC 86, 400 E. Randolph Drive,
Chicago, Illinois 60601, USA.
Further information from The Secretary,
Analytical Division, Royal Society of
Chemistry, Burlington House, London
W1V OBN.
DECEMBER 1985
Chem Asia 85
To be held from 2-5 December in
Singapore.
Further information from Chatsworth
House, 59 London Road, Twickenham
TW1 3SZ, UK.
JANUARY 1986
Membranes in Filtration Systems
To be held at UMIST, Manchester,
UK on 21 January.
Further information from The Secretary,
The Filtration Society, Department of
Chemical Engineering, University ofTech-
nology, Loughborough, Leicestershire
LEll 3TU, UK.
APRIL 1986
Fourth World Filtration Congress
To be held from 22 to 25 April 1986
in Ostend, Belgium.
Further informationfrom the Secretariat of
World Filtration Congress, IV, KVIV-
Technologisch Instituut, Jan van Rij-
swijcklaan 58, B 2018 Antwerpen, Bel-
gium.
MAY 1986
Tenth International Symposium
on Column Liquid Chromato-
graphy
To be held at the Sheraton Palace
Hotel, San Francisco, California,
USA from 18 to 23 May.
JUNE 1986
Analytica 86
To be held from 3 to 6 June 1986 at
Miinchen, FR Germany.
Further information from Miinchener
Messe-und Austellungsgesellschaft mbH,
Messegelgtnde, Postfach 12 10 09, D8000
Mnchen 12, FR Germany.
Annual Congress of the Canadian
Society of Laboratory Technolo-
gists
To be held from 15 to 20 June in St.
John's, Newfoundland, Canada.
Further informationfrom CSLT, PO Box
830, Hamilton, Ontario, Canada
L8N 3N8.
Third International Congress on
Paediatric Laboratory Medicine
To be held from 29 June to 3 July in
Bristol, UK.
Further information from Mrs Anne
Green, Department ofClinical Chemistry,
The Children's Hospital, Ladywood,
Middleway, Birmingham B16 8ET, UK.
JULY 1986
National Meeting of the AACC
To be held from 13 to 18 July in
Chicago, USA.
Further informationfrom AACC, 1725 K
Street, N.W., Suite 1010, Washington,
D.C. 20006.
SAC 86/3rd BNASS: an Interna-
tional Conference on Analytical
Chemistry
To be held from 20-26 July 1986 at
the University of Bristol, UK.
AUGUST 1986
Sixth International Congress of
Pesticide Chemistry
To be held from 10-15 August in
Ottawa, Canada.
Further information H. V. Morley, Chair-
man: Organizing Committee. The Chem-
ical Institute ofCanada, 151 Slater Street,
Suite 906, Ottawa, Ontario, Canada
K1P 5H3.
SEPTEMBER 1986
3rd African, Mediterranean and
Near East Congress of Clinical
Chemistry
To be held from 28 September to 2
October in Sevilla, Spain.
Further information from Apartado de
Correos 6209, 41080 Sevilla, Spain.
OCTOBER 1986
8th Latin American Congress for
Clinical Biochemistry/3rd Ibero-
American Congress for Clinical
Analysis/13th Colombian Con-
gress for Clinical Laboratories
To be held from 9-13 October in
Bogotgt, Columbia.
Further informationfrom Carlos Gdmez--
Vesga, Calle 45 Nos. 9-22, Ofic. 302,
Apartado (PO Box 075702), Bogotit,
Colombia.
Conference organizers should send full
details of their meeting for inclusion in
'Journal of Automatic Chemistry's'
calendar at least three months before the
event. Information to the Editor,Journal of
Automatic Chemistry, Taylor & Francis
Ltd, 4 John Street, London WC1N 2ET.
208

				

